# Effects of *Ganoderma lucidum* polysaccharide peptide ameliorating cyclophosphamide-induced immune dysfunctions based on metabolomics analysis

**DOI:** 10.3389/fnut.2023.1179749

**Published:** 2023-05-25

**Authors:** Jing Xie, Dongmei Lin, Jing Li, Tonghui Zhou, Shuqian Lin, Zhanxi Lin

**Affiliations:** ^1^College of Food Science, Fujian Agriculture and Forestry University, Fuzhou, Fujian, China; ^2^National Engineering Research Center of Juncao Technology, Fujian Agriculture and Forestry University, Fuzhou, Fujian, China; ^3^Hunan University of Humanities, Science, and Technology, Loudi, Hunan, China

**Keywords:** *Ganoderma lucidum* polysaccharide peptide, cyclophosphamide-induced mice, UPLC-MS/MS, fecal metabolome, immunomodulatory

## Abstract

*Ganoderma lucidum* polysaccharide peptide (GLPP) is one of the most abundant constituents of *Ganoderma lucidum* (*G. lucidum*), with a wide range of functional activities. The present study investigated the immunomodulatory effects of GLPP in cyclophosphamide (CTX)-induced immunosuppressive mice. The results showed that 100 mg/kg/day of GLPP administration significantly alleviated CTX-induced immune damage by improving immune organ indexes, earlap swelling rate, the index of carbon phagocytosis and clearance value, secretion of cytokines (TNF-α, IFN-γ, and IL-2), and immunoglobulin A(IgA) in the mice. Furthermore, ultra-performance liquid chromatography with mass/mass spectrometry (UPLC-MS/MS) was conducted to identify the metabolites, followed by biomarker and pathway analysis. The results showed that GLPP treatment alleviated CTX-induced alterations in the fecal metabolome profile, including arachidonic acid (AA), leukotriene D4 (LTD4), indole-3-ethanol, and formyltetrahydrofolate (CF), by reversing citric acid, malic acid, cortisol, and oleic acid. These results support the concept that GLPP exhibits immunomodulatory activity via the folate cycle, methionine cycle, TCA cycle, fatty acid biosynthesis and metabolism, glycerophospholipid metabolism, AA metabolism, and cAMP pathways. In conclusion, the results could be helpful to understand the use of GLPP to clarify the immunomodulatory mechanism and be used as immunostimulants to prevent CTX-induced side effects in the immune system.

## 1. Introduction

In recent years, the use of natural polysaccharides as immune enhancements has become a topic of intensive research (1–4). *Ganoderma lucidum* polysaccharide peptide (GLPP), as a natural polysaccharide, has been widely used in the field of immune enhancement. *Ganoderma lucidum* (*G. lucidum*), from which the GLPP is derived, is a traditional Chinese medicine that has been used for thousands of years in Asia because of its immunomodulatory, antitumor, and neuropharmacological functions (5–9). However, the attractive nature of *G. lucidum* has been proven to be related to GLPP, which is known as a significant constituent of *G. lucidum* with a molecular weight of ~5 × 10^4^ Da and a polysaccharide content of up to 87.17%, containing 16 amino acids with a total amino acid content of 5.04% (10). Previous studies have shown that GLPP has diverse bioactivities such as antioxidant, memory-enhancing, antitumor, oxidative stress-reducing, fatty liver-reducing, liver and kidney-protecting, and sleep-protecting effects, and its most important biological activity is to enhance host immunity by enhancing immune cell function and promoting the release of immune factors (11–17). The enhanced immune function of polysaccharides has benefited several areas of medical research, for instance, by reducing the side effects associated with treating cancer. Cancer is one of the major causes of death worldwide (18). Chemotherapy is one of the most common and effective methods to manage pain caused by cancer (19). This therapy leads to tumor death through its genotoxic and cytotoxic effects, which are often induced by the use of Cyclophosphamide (CTX) (20, 21). Thus, many studies have paid attention to reducing the effect of immunosuppression caused by chemotherapy (22–24). Theoretically, the enhancing immunity effect of GLPP and its underlying mechanism might be effective. However, to date, no systematic study has addressed the effects of GLPP on CTX-induced immune damage in the organism or the molecular mechanisms involved.

Metabolomics offers an alternative method for evaluating active substances associated with specific biochemical events and specific mechanisms of action (25, 26). By analyzing the quantities of endogenous metabolite changes in biological systems, the relationship between bioactive compounds' effects and biomarkers can be clarified (27). In immunology research, metabolomics is a powerful tool for exploring disease-related processes and monitoring therapeutic responses (28, 29). Therefore, metabolomics studies are necessary to clarify the mechanism by which GLPP affects immune responses.

In the present study, the immune organ indexes, the levels of immunoglobulins and cytokines in serum, mouse ear swelling (DTH Test), carbon clearance activity testing, and intestinal morphology analysis were tested and analyzed to investigate the ability of GLPP on the immune system in CTX-induced mice. All of the exams described above included immune organs, immune cells, and immune molecules of the immune system. An ultra-performance liquid chromatography-tandem mass spectrometer (UPLC-MS/MS)-based metabolomics study combined with multivariate statistical analysis was applied to identify potential biomarkers and their related pathways in CTX-induced mice after being treated with GLPP. The results suggest that GLPP was effective in protecting immune organs, enhancing the production of immune-related cytokines, and promoting intestinal health in CTX-induced mice. This effect may be due to the metabolites, such as organic acids, lipids, and carbohydrate metabolisms, mediated by GLPP, which work on immune-related metabolic pathways.

## 2. Materials and methods

### 2.1. Drugs and materials

The GLPP powder was provided by the National Engineering Research Center of JUNCAO (Fujian, China). The average molecular weight of GLPP is ~50 kDa. Cyclophosphamide (CTX, CAS:50-18-0) was purchased from Shanghai Macklin Biochemical Co., Ltd. Levamisole hydrochloride (LMS, CAS:16595-80-5), often used as a positive control drug for immunosuppression, was purchased from Beijing Solarbio Science & Technology Co., Ltd. All of the other chemicals were of analytical purity.

### 2.2. Animal and experimental design

A total of 120 male-specific pathogen-free (SPF) BALB/c mice (4–6 weeks, 20 ± 2 g) were purchased from Shanghai SLAC Laboratory Animal Co., Ltd. [Shanghai, China, Certificate number: SCXK(Hu)2017-0005]. All mice were maintained under SPF conditions (25 ± 1°C; 40–50% relative humidity; 12-h light/dark cycle; free access to food and water) for 7 days to allow for adaptation. The mice were randomized to six groups (20 mice per group): (i) the normal control group (CK), (ii) the model control CTX group (CTX), (iii) the low dose (50 mg/kg/day, BW) of the GLPP group (L-GLPP), (iv) the middle dose (100 mg/kg/day, BW) of the GLPP group (M-GLPP), (v) the high dose (200 mg/kg/day, BW) of the GLPP group (H-GLPP), and (vi) the positive control group (40 mg/kg/day, BW, LMS). During the modeling period, the CK group was intraperitoneally injected with saline, and the other groups were intraperitoneally injected with 80 mg/Kg/day CTX for 3 days. Subsequently, in the following week after modeling, the GLPP groups were intragastrically administered with the corresponding dose of GLPP, the CK group was intragastrically administered with saline, and the LMS group was intragastrically administered with LMS. All animal experiments were conducted in compliance with the national guidelines for Laboratory Animal Welfare (MOST of PR China, 2006). The protocol was approved by the Laboratory Animal Center of Fujian Medical University (permit No. FJMU IACUC 2019-0084).

### 2.3. Analysis of immune organ indexes

On the 42nd day after treatment, seven mice from every experimental group were randomly taken out and weighed. Afterward, the mice's immune organs, the spleen, and the thymus were isolated and weighed. The immune organ indexes were calculated according to the following formula: The immune organ indexes (mg/g) = weight of the spleen or thymus (mg)/body weight (g) (30).

### 2.4. Assay release of cytokines and quantity of serum immunoglobulins

The sample blood was left at room temperature to clot for 2 h and then centrifuged for 15 min at 3,000 rpm/min at 4°C (Beckman Allegra X-30R; Beckman Coulter Trading Co., Ltd., China) to separate the serum. The levels of immunoglobulin A(IgA), interleukin-2(IL-2), interferon-γ (IFN-γ), and tumor necrosis factor-α (TNF-α) in serum were analyzed using ELISA kits (ABclonal Technology Co., Ltd., Wuhan, China) according to the manufacturer's instructions.

### 2.5. Delayed-type hypersensitivity reaction (DTH)

The *in vivo* cellular immune activity was tested by the DNFB-induced delayed-type hypersensitivity (DTH) reaction. The mice were sensitized by smearing 1% (v/v) DNFB (CAS:70-34-8, Rhawn) in acetone/sesame oil (1:1) onto the shaved abdomen for 4 consecutive days. Four days later, 1% DNFB was evenly smeared on both sides of the left earlap of each mouse, and the mice in the CK group were smeared with acetone/sesame oil (1:1) without DNFB. Then, 24 h later, the ear slices (8 mm) were removed from the same part of each ear and weighed. The degree of DTH is reflected by the difference in mass between the two earpieces.

### 2.6. Detection of macrophage function by mice carbon clearance activity testing

The mice in each group were randomly selected for tail vein injection of India ink (SenBeiJia Biological Technology Co., Ltd.), with a carbon suspension of 10 μL/g body weight administered intravenously. Blood samples of 25 μL were collected at 2 and 10 min after injection of the carbon, and each blood sample was separated in a 2 mL solution of 0.1% Na_2_CO_3_. Then, the absorbance was measured with a UV-visible spectrophotometer at 550 nm. The livers and the spleens were collected and weighed separately. The index of carbon phagocytosis and clearance (α) value was calculated using the following formula (31):


$$K=\frac{\log{\text{ OD}}_{\text{1}}-\log{\text{ OD}}_{\text{2}}}{{\text{t}}_{\text{2}}-{\text{t}}_{\text{1}}}$$

$$\begin{array}{l} \alpha =\frac{{\text{W}}_{0}}{{\text{W}}_{1}+{\text{W}}_{2}}*1/3K \end{array}$$

Explanation:

*K*: Phagocytics constant.
OD_n_: Adsorbents in n time.
t_1_: Time at 2 min.
t_2_: Time at 10 min.
α: Index of carbon phagocytosis and clearance.
W_0_: The weight of the body.
W_1_: The weight of the liver.
W_2_: The weight of the spleen.

### 2.7. Histological analysis of jejunum tissues

The jejunum tissues were prepared for histological analysis using the methods described by Ying et al. (32) with some modifications. In brief, after being embedded in paraffin, samples of the jejunum tissue were fixed in 4% paraformaldehyde, then sliced at 4 μm thickness, and stained with hematoxylin and eosin (H&E). The images were taken by a light microscope (Keyence VHX-7000, 200× magnification).

### 2.8. Fecal metabolites assessment

Fecal samples (from cecum contents) were thawed on ice, and 50 mg of each sample was homogenized with 500 uL of ice-cold methanol/water (70%, v/v). The samples were vortexed for 3 min, sonicated for 10 min in an ice water bath, and then vortexed for 1 min. Then, they were centrifuged at 12,000 rpm at 4°C for 10 min. The collected supernatant was used for UPLC-MS/MS analysis.

The sample extracts were analyzed using an LC-ESI-MS/MS system (UPLC, Shim-pack UFLC SHIMADZU CBM A system, https://www.shimadzu.com/; MS, QTRAP^®^ System, https://sciex.com/). The analytical conditions were as follows: UPLC: column, Waters ACQUITY UPLC HSS T3 C18 (1.8 μm, 2.1 mm^*^100 mm); column temperature, 40°C; flow rate, 0.4 mL/min; injection volume, 2 μL; solvent system, water (0.1% formic acid): acetonitrile (0.1% formic acid); gradient program, 95:5 V/V at 0 min, 10:90 V/V at 11.0 min, 10:90 V/V at 12.0 min, 95:5 V/V at 12.1 min, and 95:5 V/V at 14.0 min. A high-resolution tandem mass spectrometer, QTRAP (AB Sciex QTRAP^®^ 6500+, UK), was used to detect metabolites eluted from the column. The ESI source operation parameters were as follows: a source temperature of 500°C; ion spray voltage (IS) of 5 KV (positive) and 4.5 KV (negative); ion source gas I (GSI), gas II (GSII), and curtain gas (CUR) were set at 55.0, 60.0, and 25.0 psi, respectively, and the collision gas (CAD) was set to high. The mass accuracy was calibrated every 20 samples, and a quality control (QC) sample was acquired from every 10 samples during acquisition.

The acquired MS raw data were converted to the mzData.xml format using the software Analyst 1.6.3 (Sciex), and the offline mass spectrometry files were used with MultiQuant software to perform the integration and correction work of the chromatographic peaks. This resulted in the obtained multivariate data matrix containing sample information, ion information [retention time (Rt) and mass-to-charge ratio (m/z)], and ion intensities. The obtained data were imported into SIMCA-P14.0 for unsupervised PCA principal component analysis and supervised OPLS-DA analysis. Candidate metabolites were considered potential biomarkers, while VIP of > 1 was a value of *p* of < 0.05. The online databases HMDB, METLIN, and KEGG were used to identify and interpret all potential marker metabolites. The metabolite content data were normalized using the *Z*-score method, and the accumulation patterns of metabolites among different samples were clustered by R software.

### 2.9. Statistical analysis

All data were presented as means ± SD. The differences between the two groups were analyzed by Student's *t*-test. Multiple group comparisons were analyzed using a one-way analysis of variance (ANOVA) with Tukey's correction. A *p*-value of < 0.05 indicated statistical significance.

## 3. Results

### 3.1. Effects of GLPP on immune organ indexes in CTX-induced mice

As summarized in Table 1, the spleen and thymus indexes were significantly decreased (*p* < 0.05) in the CTX group compared to the CK group. The spleen indexes for the L-GLPP and M-GLPP groups showed no significant changes compared with the CTX group. However, the treated group of M-GLPP or H-GLPP showed a dramatic increase of 14.4 and 19.5% for the spleen indexes (*p* < 0.01) and 94.1 and 104% for the thymus indexes of the CTX group, respectively (*p* < 0.05). In addition, there was no significant difference in thymus indexes after intervention by M-GLPP or H-GLPP compared with the LMS group.

**Table 1:** Effects of different treatment groups on immune organ indexes in CTX-induced mice (mean ± SD, *n* = 7).

**Group**	**Dose (mg/kg)**	**Immune organ weight (mg)**		**immune organ index (mg/g)**	
		**Spleen**	**Thymus**	**Spleen index**	**Thymus index**
CK	0	0.0513 ± 0.0097^*^	0.0177 ± 0.0297^**^	2.0299 ± 0.2525^*^	0.7025 ± 0.0911^**^
CTX	80	0.0386 ± 0.0658^#^	0.0078 ± 0.0373^##^	1.5919 ± 0.2662^#^	0.3215 ± 0.1463^##^
LMS	40	0.0501 ± 0.0057^*^	0.0165 ± 0.0123^**^	2.0238 ± 0.2542^**^	0.6662 ± 0.0571^**^
L-GLPP	50	0.0437 ± 0.0024^#^	0.0145 ± 0.0015^#, **^	1.7419 ± 0.0956^#^	0.5766 ± 0.0566^#, **^
M-GLPP	100	0.0439 ± 0.0065^#^	0.0150 ± 0.0133^#, **^	1.8206 ± 0.2463	0.6240 ± 0.0521^**^
H-GLPP	200	0.0478 ± 0.0058^*^	0.0164 ± 0.0018^**^	1.9025 ± 0.2121^*^	0.6559 ± 0.0846^**^

# p < 0.05 and

## p < 0.01 compared with the CK group.

* p < 0.05 and

** p < 0.01 compared with the CTX group.

### 3.2. Effects of GLPP on serum levels of immunoglobulin and cytokines in CTX-induced mice

**Figure 1:**
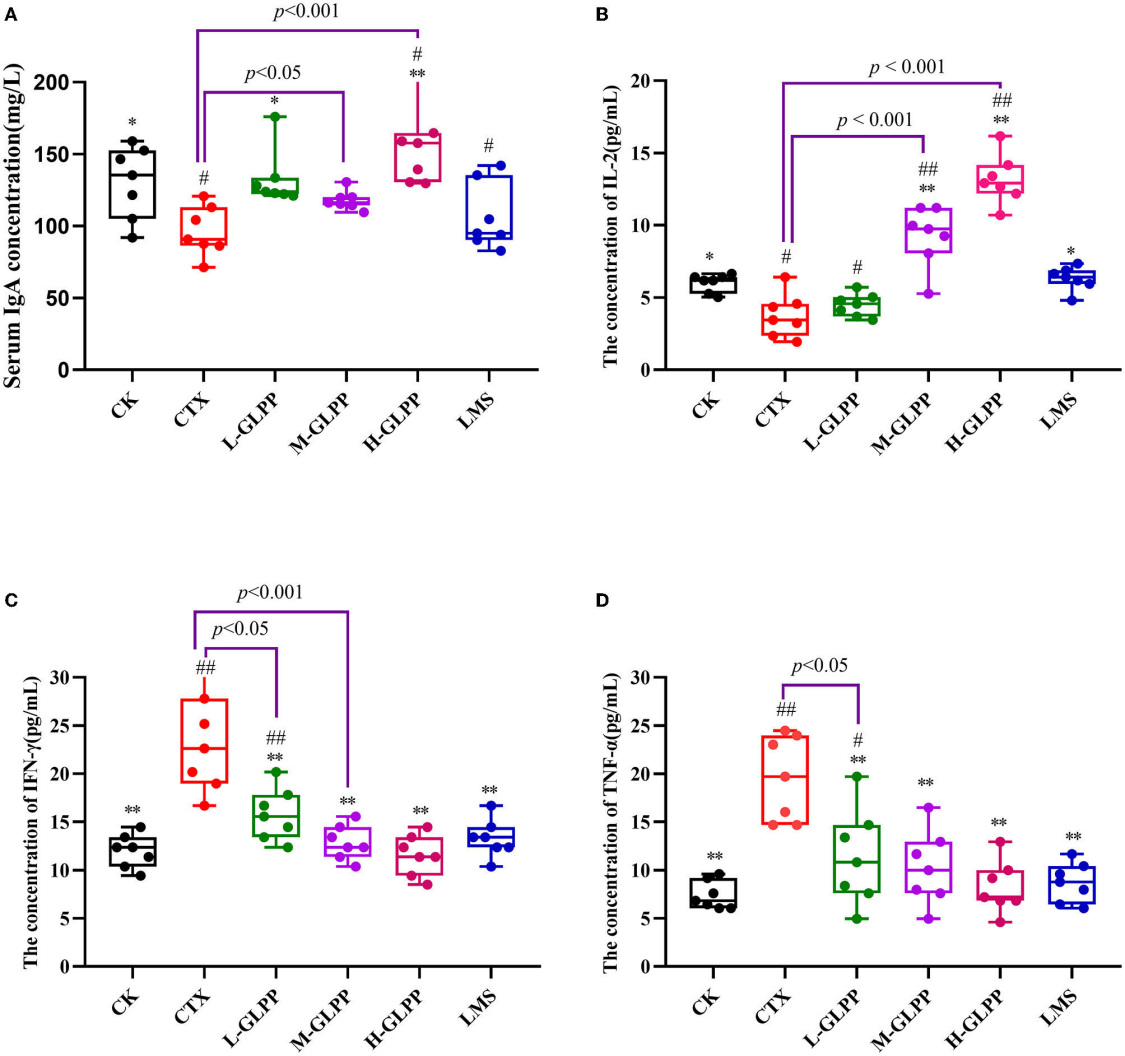
Effects of each group on the serum of immunoglobulin A **(A)**, interleukin- 2 **(B)**, interferon-γ **(C)**, and the tumor necrosis factor-α **(D)** levels in CTX-induced mice. Data are expressed as mean ± SD (*n* = 7). ^#^*p* < 0.05 and ^##^*p* < 0.01 compared with the CK group; ^*^p < 0.05 and ^**^p < 0.01 compared with the CTX group.

As shown in Figure 1A, after 6 weeks, the IgA level of mice in the CTX group was significantly lower than other groups, which was reduced by 26.08% (*p* < 0.01) compared with the CK group. After GLPP intervention, IgA concentration was increased at different levels, among which the H-GLPP group increased the most, by up to 60.6% (*p* < 0.001). However, there was no significant difference in IgA level between the LMS group and the CTX group, suggesting that the ability of GLPP intervention to improve immune response in mice may be better than that of LMS.

As presented in Figure 1B, compared with the CTX group, the levels of IL-2 in the CTX-treated mice with M-GLPP, H-GLPP, and LMS all increased significantly (*p* < 0.05). The levels of IL-2 in the M-GLPP and H-GLPP groups were up to 146 and 297%, respectively, higher than those of the CTX group (*p* < 0.001).

As shown in Figures 1C, D, compared with the CK group, TNF-α, and IFN-γ concentrations were significantly increased by 164 and 94.99% (*p* < 0.01), respectively, in the CTX group. However, the levels of these cytokines were greatly decreased in CTX-treated mice by the administration of GLPP in a dose-dependent manner (*p* < 0.01), suggesting that GLPP-treated mice could reduce the inflammatory response induced by CTX and enhance immunity by alleviating the production of pro-inflammatory factors.

### 3.3. Effects of GLPP on the DTH reaction

**Figure 2:**
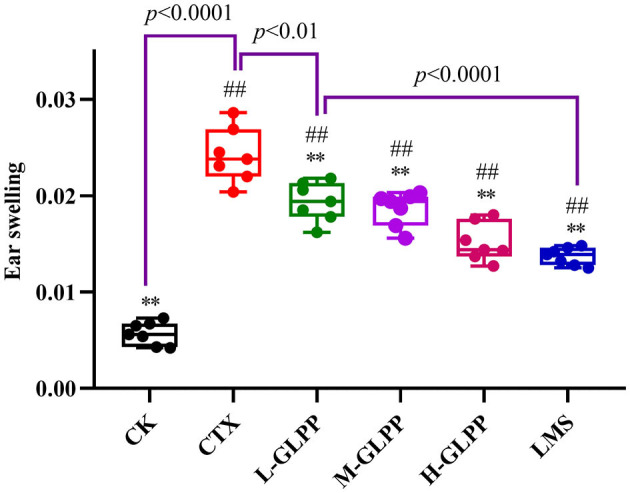
Effects of each group on ear swelling index in CTX-induced mice. Data are expressed as mean ± SD (*n* = 7). ^#^*p* < 0.05 and ^##^*p* < 0.01 compared with the CK group; ^*^p < 0.05 and ^**^p < 0.01 compared with the CTX group.

The DTH reaction is a widely used model for T-cell-mediated immune responses to contact allergens. The results are shown in Figure 2. Compared with the CK group, the earlap swelling rate was significantly increased in the CTX group, indicating that CTX damaged the cellular immunity of mice seriously. With the treatment of GLPP and LMS, the ear swelling rate decreased to varying degrees, and both treatments showed significant differences compared with the CTX group. The results showed that GLPP can increase the cellular immunity of mice.

### 3.4. Effects of GLPP on carbon clearance in mice

**Figure 3:**
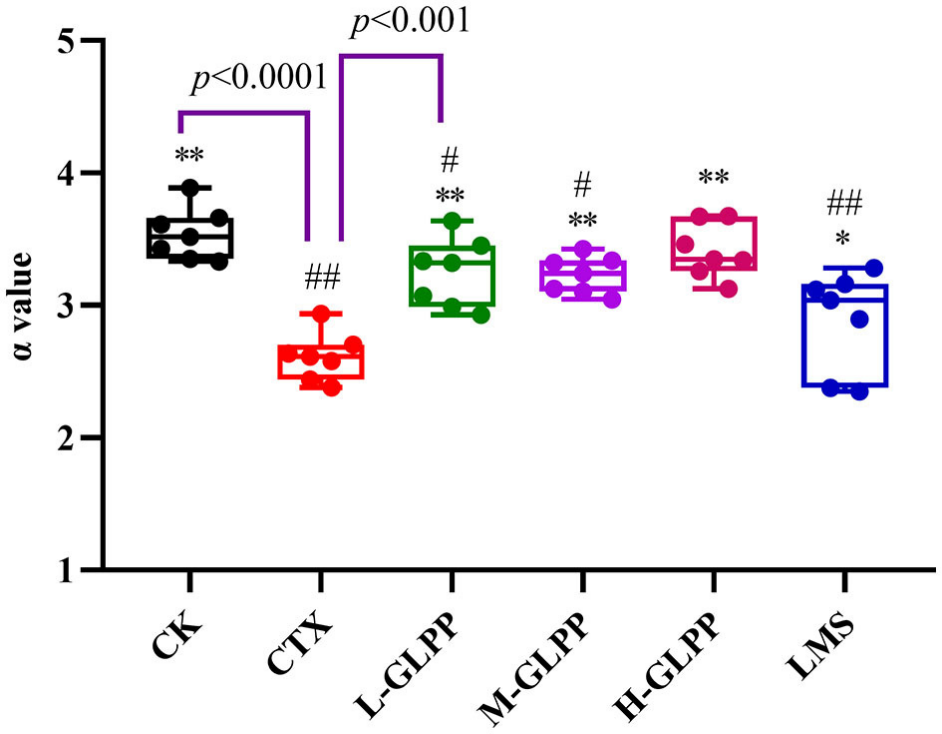
Effects of each group on the index of carbon phagocytosis and clearance (α-valve). Data are expressed as mean ± SD (*n* = 7). ^#^*p* < 0.05 and ^##^*p* < 0.01 compared with the CK group; ^*^p < 0.05 and ^**^p < 0.01 compared with the CTX group.

The phagocytic ability of mononuclear macrophages *in vivo* was measured by carbon clearance activity testing. Figure 3 shows that the α-value in the CK group was 3.54 but decreased to 2.61 (*p* < 0.01) in the CTX group. Meanwhile, the α-values in L-GLPP, M-GLPP, and H-GLPP increased to 3.25, 3.23, and 3.41, respectively, all GLPP-treated groups increased α-value significantly. The results of the α-value showed that GLPP had immunomodulatory activity.

### 3.5. Effect of GLPP on jejunum histology in mice

**Figure 4:**
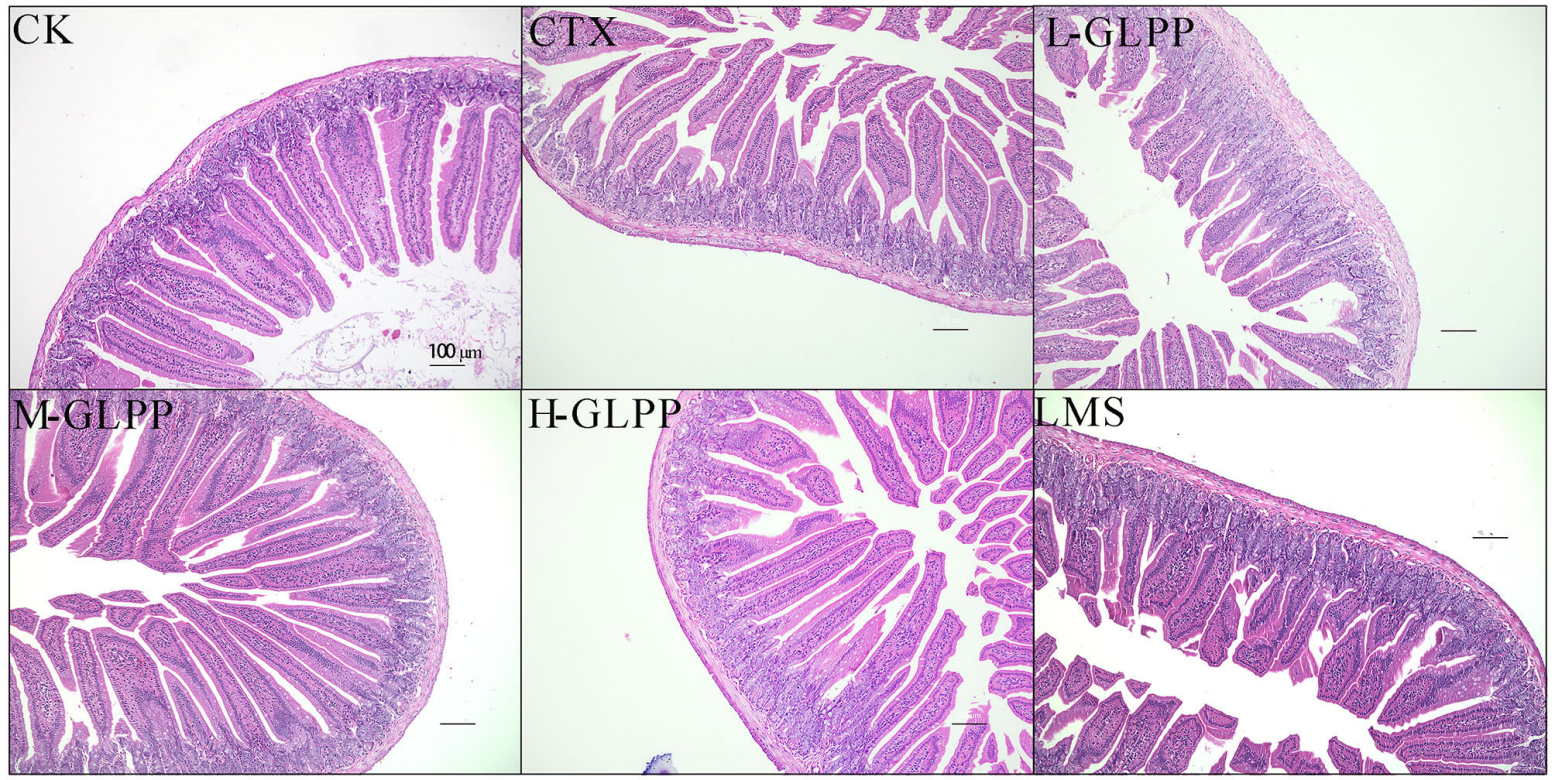
Histopathological analysis of jejunum tissue of mice in each group at 200 × magnification.

As shown in Figure 4, jejunal villi were intact and closely arranged in the tissue of the CK group under an optical microscope. However, in the tissue sections of the CTX group, serious lesions of the jejunal villi were observed, including an incomplete structure, epithelial atrophy, villus shortening, and a sparse and irregular arrangement. Compared with the tissue section of the CTX group, administration of GLPP could efficiently ameliorate CTX-treated intestinal mucosal injury, showing longer and thicker villi, a narrower villus gap, a relatively regular arrangement, and a complete structure of villi.

### 3.6. GLPP treatments alter fecal metabolites in CTX-induced mice

**Figure 5:**
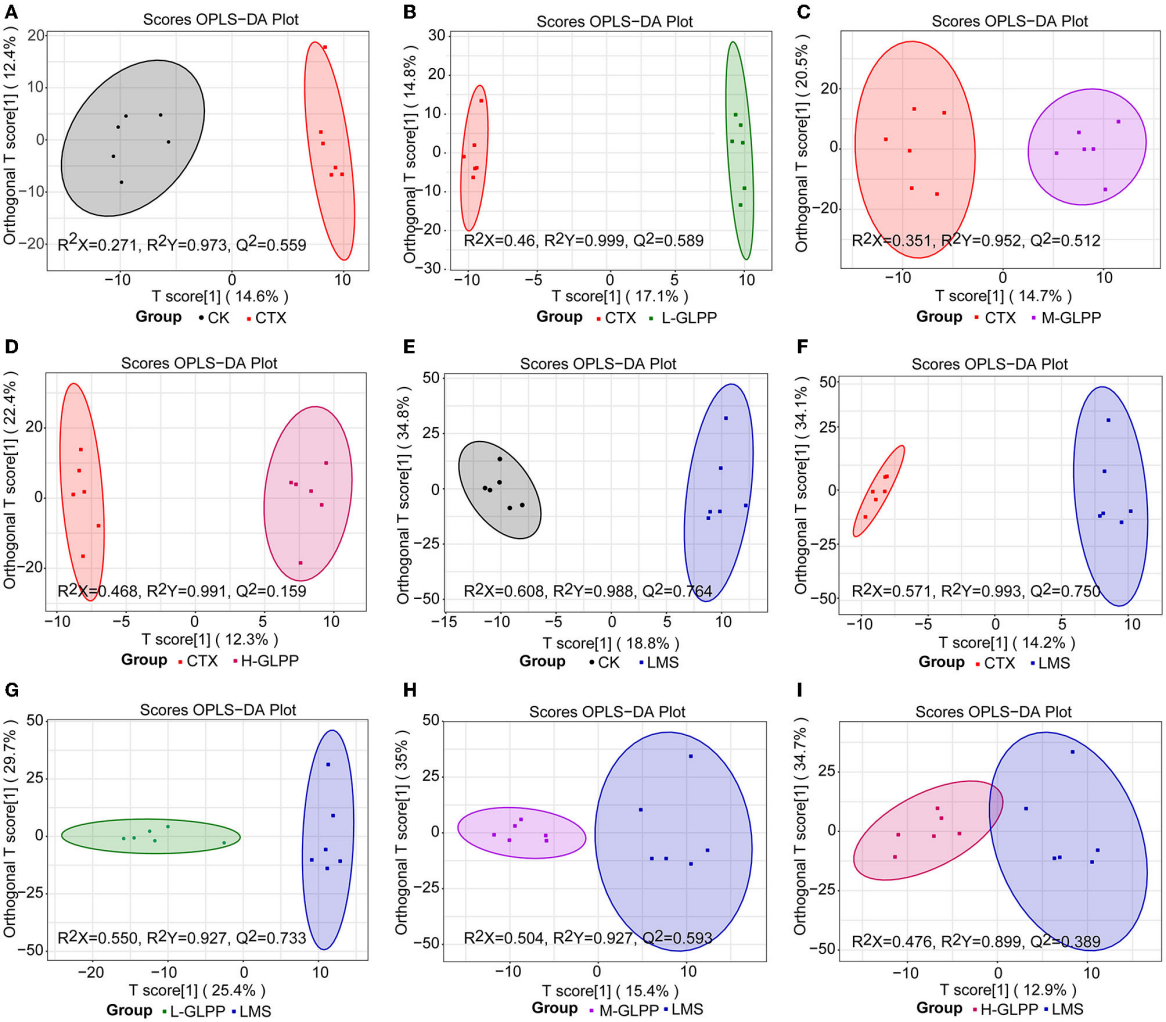
The OPLS-DA score plot of the distinct metabolites. **(A–D)** are representations of the CTX group vs. the CK group, the CTX group vs. the L-GLPP group, the CTX group vs. the M-GLPP group, the CTX group vs. the H-GLPPgroup, respectively; **(E, G–I)** are representations of the LMS group vs. the CK group, the LMS group vs. the L-GLPP group, the LMS group vs. the M-GLPP group, the LMS group vs. the H-GLPP group, respectively; **(F)** is a representation of the CTX group vs. the LMS group.

Gut immunity is an important constituent of the immune system, and metabolites play an important role in immunometabolism. Hence, the UPLC-QTrap-MS/MS method was used to obtain metabolic profiles of cecum contents in different experimental groups. There were 592 metabolites identified in the negative and positive modes, respectively. To eliminate the effect of mixed variables and to evaluate the statistical significance of the signals, the OPLS-DA model was established to analyze the difference between the CK group, the CTX group, the GLPP-treated group, and the LMS group. Figures 5A–D, F show that the CTX group was significantly separated from the CK group, the GLPP-treated group, and the LMS group (*R*^2^X = 0.271, *R*^2^Y = 0.973, *Q*^2^ = 0.559; *R*^2^X = 0.46, *R*^2^Y = 0.999, *Q*^2^ = 0.589; *R*^2^X = 0.351, *R*^2^Y = 0.952, *Q*^2^ = 0.512; *R*^2^X = 0.468, *R*^2^Y = 0.991, *Q*^2^ = 0.159; *R*^2^X = 0.571, *R*^2^Y = 0.993, *Q*^2^ = 0.750), indicating that CTX treatment caused changes in gut metabolites, while Figures 5E, G–I show relatively close clustering between the LMS group the GLPP-treated group, which, to some extent, indicated that the metabolites in mice treated with GLPP were closer to mice treated with LMS without significant differences (*R*^2^X = 0.608, *R*^2^Y = 0.988, *Q*^2^ = 0.764; *R*^2^X = 0.550, *R*^2^Y = 0.927, *Q*^2^ = 0.733; *R*^2^X = 0.504, *R*^2^Y = 0.927, *Q*^2^ = 0.593; *R*^2^X = 0.476, *R*^2^Y = 0.899, *Q*^2^ = 0.389). *Q*^2^ represents the predictive ability of the model, and a model can be considered valid if *Q*^2^ is > 0.5.

### 3.7. Characteristic features of fecal metabolites in CTX-induced mice

To identify significantly changed metabolite features with VIP ≥ 1, FC ≤ 0.05, or FC ≥ 2 were identified as differentially expressed metabolites. The differential metabolites between different experimental groups are shown in Tables 2–4.

**Table 2:** The differentially expressed metabolites between control and CTX treat mice (trends CK vs. CTX).

**No**	**KEGG ID**	**Compounds**	**Mode**	***P*-value**	**Class**	**VIP**	**FC**	**Trends**
1	C02640	3-Methyl-1-butylamine	Positive	0.0315	Amines	1.7046	0.4658	↓
2	C00158	Citric acid	Negative	0.0411	Amino acid metabolomics	1.6794	0.4201	↓
3	C00497	D-(+)-Malic acid	Negative	0.0815	Amino acid metabolomics	1.7524	0.3801	↓
4	C00086	Urea	Positive	0.0494	Amino acid metabolomics	2.0128	0.4109	↓
5	C00955	Indole-3-ethanol	Negative	0.0535	Benzene and substituted derivatives	1.6344	0.2629	↓
6	C05608	P-Coumaraldehyde	Positive	0.0618	Benzene and substituted derivatives	1.0139	0.4920	↓
7	C00092	D-Glucose 6-phosphate	Negative	0.0696	Carbohydrate metabolomics	1.2323	0.3515	↓
8	C00117	Ribulose-5-phosphate	Negative	0.0808	Carbohydrate metabolomics	1.3512	0.4149	↓
9	C00231	D-Xylulose 5-phosphate	Negative	0.0391	Carbohydrate metabolomics	1.5246	0.3233	↓
10	C00352	D-Glucosamine 6-phosphate	Negative	0.0475	Carbohydrate metabolomics	1.6359	0.4233	↓
11	C00295	Orotic acid	Negative	0.0648	CoOthersEnzyme Factor and vitamin	1.9785	0.2933	↓
12	C00627	Pyridoxine 5′-phosphate	Positive	0.0110	CoOthersEnzyme Factor and vitamin	2.1019	0.4151	↓
13	C16677	4-Hydroxyretinoic acid	Positive	0.0868	CoOthersEnzyme Factor and vitamin	1.7262	0.4147	↓
14	C00735	Cortisol	Negative	0.0833	Lipids	1.6728	0.4779	↓
15	C00249	Hexadecanoic acid (C16:0)	Negative	0.0376	Lipids_Fatty acids	1.8592	0.4845	↓
16	C00093	Glycerol 3-phosphate	Negative	0.0815	Lipids_Fatty acids	1.4040	0.3138	↓
17	C00105	Uridine 5-monophosphate	Negative	0.0464	Nucleotide metabolomics	1.5912	0.4696	↓
18	C00029	UDP-glucose	Negative	0.0363	Nucleotide metabolomics	1.7178	0.3588	↓
19	C07480	Theobromine	Positive	0.0161	Nucleotide metabolomics	2.1335	0.4351	↓
20	C01367	3'-Aenylic acid	Positive	0.0190	Nucleotide metabolomics	1.6223	0.4562	↓
21	C00942	Guanosine 3',5′-cyclic monophosphate	Positive	0.1094	Nucleotide metabolomics	1.6403	0.4667	↓
22	C03406	Argininosuccinic acid	Negative	0.0715	Organic acid and its derivatives	1.3314	0.3591	↓
23	C00219	AA [5Z,8Z,11Z,14Z-eicosatetraenoic acid]	Negative	0.0046	Oxidized lipid	1.7518	0.1803	↓
24	C00234	10-Formyl-Thf	Positive	0.0407	Pteridines and derivatives	1.9721	0.2718	↓
25	C00097	L-Cysteine	Negative	0.1802	Amino acid metabolomics	1.1074	2.0942	↑
26	-	2-n-Pentylfuran	Negative	0.0080	Heterocyclic compound	1.2530	2.3136	↑
27	-	7-ketodeoxycholic acid	Negative	0.1500	Lipids	1.2191	4.1434	↑
28	C00712	Oleate	Positive	0.0502	Lipids	1.7902	2.0922	↑
29	-	Hexadecanamide	Positive	0.0091	Lipids_Fatty Acids	2.1313	2.8266	↑
30	C19670	Oleamide	Positive	0.0480	Lipids_Fatty Acids	1.7908	2.0690	↑
31	C00170	5′-Deoxy-5′-(Methylthio) adenosine	Positive	0.0490	Nucleotide metabolomics	2.0057	4.5001	↑
32	C00493	Shikimic acid	Negative	0.1124	Organic acid and its derivatives	1.1184	5.2473	↑
33	C19524	Succinic anhydride	Negative	0.2110	Organic acid and its derivatives	1.2435	2.1238	↑
34	C00852	Chlorogenic acid	Positive	0.1194	Organic acid and its derivatives	1.3103	4.1390	↑
35	-	(±)12-HETE [(±)12-hydroxy-5Z,8Z,10E,14Z-eicosatetraenoic acid]	Negative	0.3186	Oxidized lipid	1.1172	2.2846	↑
36	C00315	Spermidine	positive	0.0765	Polyamine	1.2854	5.7341	↑

↓ means the corresponding compounds was be down-regulated, and the ↑ was be up-regulated.

The results in Table 2 show that, compared with the CK group, 36 differentially expressed metabolites were identified in the CTX group, with 12 upregulated metabolites and 24 downregulated metabolites. Spermidine, Shikimic acid, 5′-deoxy-5′-(methylthio) adenosine, 7-keto deoxycholic acid, chlorogenic acid, hexadecanamide, 2-n-Pentylfuran, (±)12-HETE [(±)12- hydroxy-5Z,8Z,10E,14Z-eicosatetraenoic acid (12-Hete)], succinic anhydride, and L-cysteine were significantly upregulated in the CTX group, where arachidonic acid (AA), indole-3-ethanol, formyltetrahydrofolate (CF), lactic acid, glycerophosphate, D-xylulose-5 phosphate, D-glucose-6-phosphate, UDP glucose, argininosuccinic acid, and D-(+)-malic acid were significantly downregulated.

The results in Table 3 show that, compared with the CTX group, 30 metabolites were significantly altered in the GLPP group, with six upregulated metabolites and 24 downregulated metabolites. In the GLPP group, LTD4, palmitoylcarnitine, indole-3-ethanol, 2,5-dimethylfuran, P-acetaminophen-β-D-glucosinolate, and hexadecane (16:0) were significantly upregulated, while hexanoyl glycine, argininosuccinic acid, D-xylulose-5-phosphate, D-glucose-6-phosphate, nicotinamide, glycerophosphate, ribulose-5-phosphate, 3-hydroxybutyric acid, L-lactic acid, malonic acid, N-acetyl-L-alanine, DL-2-amino succinic acid, O-acetylcarnitine, and UDP glucose were significantly downregulated.

**Table 3:** The differentially expressed metabolites between CTX-treated and GLPP-treated mice (trends CTX vs. GLPP).

**No**	**KEGG ID**	**Compounds**	**Mode**	***P*-value**	**Class**	**VIP**	**FC**	**Trends**
1	C00519	2-Aminoethanesulfinic acid	Negative	0.1490	Organic acid and its derivatives	1.2395	0.4172	↓
2	C00300	Creatine	Negative	0.0347	Organic acid and its derivatives	1.8919	0.4469	↓
3	C01124	18-Hydroxycorticosterone	Positive	0.0081	Lipids	1.1993	0.4440	↓
4	-	Hexanoyl glycine	Negative	0.3009	Amino acid metabolomics	1.4921	0.0462	↓
5	C00092	D-Glucose 6-phosphate	Negative	0.1042	Carbohydrate metabolomics	1.5477	0.2087	↓
6	C05984	2-Hydroxybutanoic acid	Negative	0.0695	Organic acid and its derivatives	1.5273	0.4891	↓
7	C01089	3-Hydroxybutyrate	Negative	0.0387	Organic acid and its derivatives	1.3844	0.2999	↓
8	C00186	L-Lactic acid	Negative	0.0313	Organic acid and its derivatives	1.7889	0.3573	↓
9	C00383	Malonicacid	Negative	0.0221	Organic acid and its derivatives	2.0006	0.3873	↓
10	C00117	Ribulose-5-phosphate	Negative	0.1080	Carbohydrate metabolomics	1.5571	0.2792	↓
11	C03406	Argininosuccinic acid	Negative	0.1178	Organic acid and its derivatives	1.3390	0.0946	↓
12	C00029	UDP-glucose	Negative	0.2005	Nucleotide metabolomics	1.1835	0.4161	↓
13	C00093	Glycerol 3-phosphate	Negative	0.0883	Lipids_Fatty acids	1.5949	0.2523	↓
14	C00362	deoxyguanosine 5′-monophosphate (dGMP)	Negative	0.0893	Nucleotide metabolomics	1.2257	0.4653	↓
15	C00231	D-Xylulose 5-phosphate	Negative	0.1111	Carbohydrate metabolomics	1.2536	0.1776	↓
16	-	17(18)-EpETE [(±)17,18-epoxy-5Z,8Z,11Z,14Z-eicosatetraenoic acid]	Negative	0.0117	Oxidized lipid	1.9873	0.4768	↓
17	C00153	Nicotinamide	Positive	0.2734	CoOthersEnzyme Factor and vitamin	1.1772	0.2416	↓
18	-	Dl-2-Aminooctanoic acid	Positive	0.0412	Organic acid and its derivatives	1.2803	0.4159	↓
19	-	2-(Dimethylamino)Guanosine	Positive	0.0829	Nucleotide metabolomics	1.5560	0.4652	↓
20	C02571	Acetyl-L-carnitine	Positive	0.0610	Camitine	1.8496	0.4160	↓
21	-	Hexadecanamide	Positive	0.0247	Lipids_Fatty acids	1.8146	0.4869	↓
22	-	N-Acetyl-L-alanine	Positive	0.0177	Organic acid and its derivatives	1.9424	0.4078	↓
23	C19670	Oleamide	Positive	0.0286	Lipids_Fatty acids	1.7607	0.4237	↓
24	C00712	Oleate	Positive	0.0325	Lipids	1.7337	0.4277	↓
25	C00955	Indole-3-ethanol	Negative	0.0846	Benzene and substituted derivatives	1.5440	3.2051	↑
26	-	Palmitoylcarnitine	Positive	0.0935	Camitine	1.3114	5.6402	↑
27	C05951	LTD4 [5S-hydroxy-6R-(S-cysteinylglycinyl)-7E,9E,11Z,14Z-eicosatetraenoic acid]	Positive	0.0048	Lipids_Fatty acids	2.0957	9.3067	↑
28	C00249	Hexadecanoic acid (C16:0)	Negative	0.0841	Lipids_Fatty acids	1.2329	2.1075	↑
29	-	Acetaminophen glucuronide	Negative	0.0586	Carbohydrate metabolomics	1.1558	2.5166	↑
30	-	2,5-Dimethylfuran	Negative	0.0176	Heterocyclic compound	1.8323	2.5190	↑

↓ means the corresponding compounds was be down-regulated, and the ↑ was be up-regulated.

The results in Table 4 show that, compared with the CTX group, 64 metabolites were significantly altered in the LMS group, with 25 upregulated metabolites and 39 downregulated metabolites. Metabolites such as LTD4, sulfamethazine, inositol, 2-aminoethanesulfinic acid, creatine, 12-hete, and indole-3-ethanol were significantly upregulated in the LMS group, L-sepiapterin, 3-methyl-1-butylamine, 3-hydroxypicolinic acid, 2,4-dihydroxybenzoic acid, protocatechuic acid, mexiletine, 3-(3-Hydroxyphenyl)-3-hydroxypropanoic acid, hydroquinone, farnesene, and all-trans-13,14-dihydroretinol were significantly downregulated.

**Table 4:** The differentially expressed metabolites between CTX-treated and imidazole hydrochloride-protected mice (trends CTX vs. LMS).

**No**	**KEGG ID**	**Compounds**	**Mode**	***P*-value**	**Class**	**VIP**	**FC**	**Trends**
1	C01124	18-Hydroxycorticosterone	Positive	0.0203	Lipids	1.2162	0.4465	↓
2	C02640	3-Methyl-1-butylamine	Positive	0.0065	Amines	2.6396	0.0004	↓
3	C00019	S-Adenosyl-L-methionine	Positive	0.0016	Amino acid metabolomics	2.1738	0.3450	↓
4	C10833	Syringic acid	Negative	0.0125	Benzene and substituted derivatives	2.2276	0.2766	↓
5	-	2,4-Dihydroxybenzoic acid	Negative	0.1155	Benzene and substituted derivatives	1.5745	0.0008	↓
6	C05608	P-Coumaraldehyde	Positive	0.1138	Benzene and substituted derivatives	1.0089	0.2899	↓
7	C00628	2,5-Dihydroxy benzoic acid	Negative	0.1277	Benzoic acid and its derivatives	1.4591	0.3005	↓
8	C09276	MARMESIN	Negative	0.1385	Carbohydrate metabolomics	1.6332	0.2580	↓
9	C00314	Pyridoxine	Positive	0.0072	CoOthersEnzyme Factor and vitamin	2.2375	0.2848	↓
10	C15492	All-Trans-13,14-Dihydroretinol	Positive	0.0738	CoOthersEnzyme Factor and vitamin	1.0514	0.1429	↓
11	C00899	11-Cis-Retinol	Positive	0.1579	CoOthersEnzyme Factor and vitamin	1.0394	0.1671	↓
12	C02538	estrone 3-sulfate	Negative	0.1321	Hormones	1.1713	0.1911	↓
13	-	3-Indolepropionic acid	Negative	0.0372	Indole and its derivatives	1.6104	0.4558	↓
14	C05635	5-Hydroxyindole-3-acetic acid	Positive	0.0450	Indole and its derivatives	1.9109	0.1765	↓
15	C14827	9-Hpode	Negative	0.0000	Lipids	2.1352	0.3364	↓
16	C09665	Farnesene	Positive	0.0453	Lipids_Fatty acids	1.4694	0.1214	↓
17	C00016	Flavin adenine dinucleotide	Negative	0.0088	Nucleotide metabolomics	1.8108	0.4781	↓
18	C00301	ADP-ribose	Negative	0.0566	Nucleotide metabolomics	1.7182	0.4684	↓
19	C00003	Nicotinic acid adenine dinucleotide	Positive	0.0004	Nucleotide metabolomics	2.2270	0.3923	↓
20	C00170	5′-Deoxy-5′-(Methylthio) adenosine	Positive	0.0513	Nucleotide metabolomics	1.9937	0.2360	↓
21	C02678	Dodecanedioic acid	Negative	0.0039	Organic acid and its derivatives	1.9213	0.4209	↓
22	C05629	Hydrocinnamic acid	Negative	0.0062	Organic acid and its derivatives	2.1962	0.2163	↓
23	C05607	L-3-Phenyllactic acid	Negative	0.0014	Organic acid and its derivatives	2.4211	0.2128	↓
24	C05942	Pyrrole-2-carboxylic acid	Negative	0.0015	Organic acid and its derivatives	2.1289	0.3422	↓
25	C11457	3-(3-Hydroxyphenyl)propionate acid	Negative	0.0626	Organic acid and its derivatives	2.1878	0.1117	↓
26	-	3-(3-Hydroxyphenyl)-3-hydroxypropanoic acid	Negative	0.1048	Organic acid and its derivatives	1.3827	0.2310	↓
27	C00331	Indole-3-pyruvic acid	Negative	0.0887	Organic acid and its derivatives	1.3800	0.4682	↓
28	C02946	4-Acetamidobutyric acid	Positive	0.0014	Organic acid and its derivatives	1.0652	0.4041	↓
29	C02470	Xanthurenic acid	Positive	0.0293	Organic acid and its derivatives	1.5984	0.1039	↓
30	-	(±)5-HEPE [(±)-5-hydroxy-6E,8Z,11Z,14Z,17Z-eicosapentaenoic acid]	Negative	0.0009	Oxidized lipid	1.8348	0.4137	↓
31	C14826	12,13-EpOME [(±)12(13)epoxy-9Z-octadecenoic acid]	Negative	0.0309	Oxidized lipid	1.5653	0.4997	↓
32	C18166	Enterodiol	Negative	0.0464	Phenols and its derivatives	1.6577	0.2749	↓
33	C00230	Protocatechuic acid	Negative	0.0094	Phenols and its derivatives	2.2951	0.0410	↓
34	C00530	Hydroquinone	Negative	0.0111	Phenols and its derivatives	2.2134	0.1181	↓
35	C01987	2-Aminophenol	Positive	0.0147	Phenols and its derivatives	2.0912	0.4365	↓
36	C00315	Spermidine	Positive	0.0801	Polyamine	1.4144	0.1873	↓
37	C00835	L-Sepiapterin	Positive	0.0590	Pteridines and derivatives	1.8702	0.0001	↓
38	C18620	3-Hydroxypicolinic acid	Negative	0.0279	Pyridine and pyridine derivatives	1.9303	0.0007	↓
39	-	6-Methylnicotinamide	Positive	0.0039	Pyridine and pyridine derivatives	1.9798	0.4688	↓
40	C00955	Indole-3-ethanol	Negative	0.0550	Benzene and substituted derivatives	1.8475	3.2618	↑
41	-	Palmitoylcarnitine	Positive	0.1191	Camitine	1.4515	27.9250	↑
42	C05951	LTD4 [5S-hydroxy-6R-(S-cysteinylglycinyl)-7E,9E,11Z,14Z-eicosatetraenoic acid]	Positive	0.0145	Lipids_Fatty acids	1.5585	9.5833	↑
43	C00519	2-Aminoethanesulfinic acid	Negative	0.2670	Organic acid and its derivatives	1.1016	4.4528	↑
44	C00300	Creatine	Negative	0.1871	Organic acid and its derivatives	1.0104	3.7274	↑
45	C02291	L-Cystathionine	Positive	0.2922	Amino acid metabolomics	1.1804	2.3505	↑
46	C19910	N-Acetylneuraminic acid	Positive	0.0192	Amino acid metabolomics	2.1258	2.1336	↑
47	C19530	Sulfamethazine	Positive	0.1898	Benzene and substituted derivatives	1.0786	7.8538	↑
48	C05465	Taurochenodesoxycholic acid	Negative	0.2748	Bile acids	1.0286	2.8893	↑
49	C00318	L-Carnitine	Positive	0.1985	Camitine	1.3389	2.6469	↑
50	-	DL-Carnitine	Positive	0.2306	Camitine	1.1639	2.6385	↑
51	-	Inositol	Negative	0.2277	Carbohydrate metabolomics	1.0051	4.6744	↑
52	C04256	N-Acetylglucosamine 1-phosphate	Negative	0.1219	Carbohydrate metabolomics	1.3489	2.2020	↑
53	C00670	Sn-Glycero-3-phosphocholine	Positive	0.2671	Cholines	1.1943	5.8027	↑
54	-	12-Hete	Negative	0.2799	Lipids	1.3129	3.7202	↑
55	-	PAF C-16	Positive	0.0754	Lipids	1.2682	3.1825	↑
56	C00670	Glycerophosphatidylcholine	Positive	0.2362	Lipids	1.1889	4.5492	↑
57	-	Lysope 18:0	Negative	0.0705	LipidsOthersPhospholipid	1.3304	2.6259	↑
58	C04230	Lysopc 16:0	Positive	0.1981	LipidsOthersPhospholipid	1.0576	2.2224	↑
59	C04230	Lysopc 18:0	Positive	0.0795	LipidsOthersPhospholipid	1.2882	3.2127	↑
60	C04230	Lysopc 20:1	Positive	0.2371	LipidsOthersPhospholipid	1.0380	2.6898	↑
61	C04230	Lysopc 17:0	Positive	0.1647	LipidsOthersPhospholipid	1.1082	2.6560	↑
62	C07480	Theobromine	Positive	0.0442	Nucleotide metabolomics	1.2456	2.0437	↑
63	-	1-Methylxanthine	Positive	0.0275	Nucleotide metabolomics	1.6463	2.5244	↑
64	C00245	2-Aminoethanesulfonic acid	Negative	0.2032	Organic acid and its derivatives	1.3806	2.0101	↑

↓ means the corresponding compounds was be down-regulated, and the ↑ was be up-regulated.

As described above, metabolite metabolism disorders caused by CTX, such as citric acid, D-(+)-malic acid, shikimic acid, succinic anhydride, chlorogenic acid, AA, CF, L-cysteine, and cortisol, were effectively returned to normal under the treatment of GLPP. Indole-3-ethanol, palmitoylcarnitine, acetaminophen glucuronide, and hexadecane (16:0) were significantly upregulated in the GLPP group. Note that some lipid metabolites expressed differences between the GLPP group and the LMS group, such as lysolecithin phosphatidylcholines (LPCs), glycerophosphatidylcholine, carnitines, and 6-methylnicotinamide, which were significantly upregulated in the LMS group, where O-acetylcarnitine, hexadecanamide, and oleamide were significantly down-regulated in the GLPP group; therefore, it can be concluded that treatment with GLPP or LMS had different metabolic pathways for CTX-induced immune damage.

### 3.8. Major differential metabolic pathway analysis

**Figure 6:**
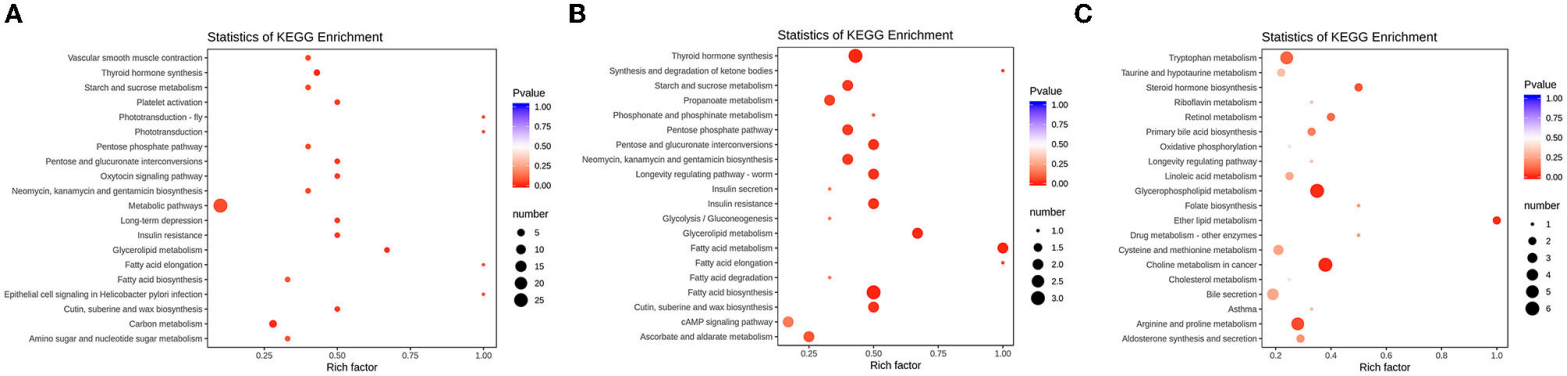
Bubble plots of enrichment of differentially expressed metabolite in KEGG pathways. **(A)** KEGG pathway enrichment of the differences between the CK group and the CTX group; **(B)** KEGG pathway enrichment of the differences between the CTX group and the GLPP group; **(C)** KEGG pathway enrichment of the differences between the CTX group and the LMS group; The horizontal coordinate represents the rich factor of each pathway, and the vertical coordinate represents pathway name in KEGG, the size of the dot represents the number of enriched metabolites. The color of the dot represents *p*-value, the darker of red dot means higher of enrichment.

To further find the pathways of differential metabolites, KEGG was used to perform a pathway enrichment analysis (Figure 6). It was shown that CTX, GLPP, and LMS mediated different pathways, among which the CTX group significantly affected four metabolic pathways, including one-carbon metabolism, central carbon metabolism (CCM), thyroid hormone synthesis, and amino acid metabolism. The GLPP group significantly affected the pentose phosphate pathway (PPP) and the tricarboxylic acid cycle (TCA) pathway, one-carbon metabolism, fatty acid biosynthesis and metabolism, propionate metabolism, glycerophospholipid metabolism, and the cAMP signaling pathway. In contrast, the LMS group mainly affected choline metabolism, glycerophospholipid metabolism, ether lipid metabolism, amino acid metabolism (arginine and proline metabolism, tryptophan metabolism, cysteine and methionine metabolism, and tyrosine metabolism), and the AMPK signaling pathway.

Metabolites such as L-cysteine, citric acid, ribulose-5-phosphate, D-xylulose-5-phosphate, and CF were involved in CCM. UDP glucose and glycerol phosphate were involved in glycerophospholipid metabolism. While citric acid, D-(+)-malic acid, urea, indole-3-ethanol, D-glucose-6-phosphate, and D-glucosamine-6-phosphate were the metabolites mainly involved in the TCA cycle. Multiple metabolic pathways were pointed out as being associated with arachidonic acid (AA).

## 4. Discussion

*Ganoderma lucidum* polysaccharide peptide has a wide range of pharmacological activities. However, its role in ameliorating CTX-induced immunosuppression has not yet been studied. Hence, in the present study, we used the CTX-induced immunosuppression model and studied the effect and potential mechanisms of GLPP on immunomodulatory properties. Many researchers have investigated the immunomodulatory effect of dietary polysaccharides on immune defense pathways (33, 34). It was found that CTX can reduce the thymus and spleen indexes in mice. Our results found that the thymus and spleen indexes for the CTX-treated group decreased significantly (*p* < 0.05) compared with the CK group, which was consistent with previous studies. We also found that the treatment with GLPP showed a dramatic increase in the spleen and thymus indexes, suggesting that GLPP increased host immune function by stimulating immune organ development.

Cytokine is a low molecular weight, a soluble protein with extensive biological activities that have many functions, such as regulating innate and adaptive immunity, blood cell generation, cell growth, and repair of damaged tissues (35). It was found that polysaccharides can reduce CTX-induced immune injury by activating immune cells and upregulating cytokine levels (36). *Auricularia auricula* polysaccharides improve CTX-induced immunosuppression in mice by increasing serum IFN-γ, IL-2, IL-4, IL-10, and TNF-α levels (37). *Cordyceps sinensis* polysaccharide regulates the production of IL-17, IL-21, and TGF by immune cells in the small intestine to reduce injury caused by CTX (32). The present study found that GLPP could promote serum IL-2, TNF-α, and IFN-γ levels secretion to regulate systemic immune function.

The intestinal epithelium acts as a physical barrier and performs intestinal mucosal immune effects by recognizing and communicating with the microbiota and immune cells (38). Previous research showed that, after CTX treatment, the villus and crypt structures of mice were seriously damaged, while *Sargassum fusiform* and *Cordyceps sinensis* polysaccharides could improve the villus length and crypt depth of mice with CTX-induced intestinal injury (4, 39). Consistent with previous research results, GLPP intervention alleviated this damage in a dose-dependent manner.

Hence, GLPP treatment can compensate for the immune damage caused by CTX induction, but the specific metabolic pathways by which GLPP regulates body immunity have never been revealed. With metabolomics analysis, the present study found that GLPP mainly used one-carbon metabolism, the TCA cycle, the glycerophospholipid metabolism, AA, and cAMP signaling pathways to compensate for the immune damage caused by CTX induction, while the LMS group regulated immune damage mainly through choline metabolism, glycerophospholipid metabolism, and amino acid metabolism. Therefore, there were significant differences in the metabolic pathways and mechanisms between GLPP and LMS interventions in immunity.

One-carbon metabolism includes the folate cycle, the methionine cycle, and the transsulfuration pathway (40). CF, an important member of one-carbon metabolism, is a derivative of folic acid in the body (41). The present study showed that the expression level of CF was downregulated in the CTX group and normalized after treatment by GLPP and LMS. In the methionine cycle, the one-carbon unit can be used for homocysteine remethylation to regenerate methionine (42, 43). In the present study, L-cysteine, which is involved in the methionine cycle, was significantly upregulated in the CTX group, and L-cysteine and 5-deoxy-5-methylthioadenosine were normalized after the treatment of GLPP. Thus, it can be speculated that GLPP interferes with the immunity of the organism by mediating the folate cycle and methionine cycle in one-carbon metabolism. From this pathway, the effect of GLPP is superior to that of LMS, which could restore the immunosuppressed mice to normality more effectively.

CCM includes glycolytic pathways (EMP), PPP, and TCA (44). The TCA cycle is the hub and ultimate catabolic pathway linking carbohydrate, protein, and lipid metabolism, which act as signaling molecules to regulate immune cell function and intervene in immune metabolism (45, 46). As shown in the present study, citric acid and malic acid were significantly downregulated in the CTX group and returned to normal after the treatment of GLPP and LMS. This was consistent with the metabolites and metabolic pathways screened in CTX-immunosuppressed mice with *Astragalus* and *Ginseng* and their combinations (47). The LMS group modulated immunity by downregulating ribulose-5-phosphate and D-xylose-5-phosphate, interfering with the glucuronic acid cycle and the PPP pathway. Hence, it can be speculated that GLPP is engaged in the body's energy metabolism through the regulation of citric and malic acids in the TCA cycle, while LMS through the regulation of the glucuronide cycle and the PPP pathway. GLPP and LMS can regulate the body's immunity, but the mechanisms are different and need further investigation.

Lipid metabolism mainly includes fatty acid and cholesterol metabolism related to tumor immunity (48, 49). Previous research showed that inhibiting lipid synthesis and metabolic signaling relies on an effective antitumor immune response to enhance the immunotherapeutic effects of PD-1 inhibitors on killer T lymphocytes (50, 51). It was demonstrated that *G. lucidum* spore powder could inhibit PD-1 expression by suppressing the phosphorylation level of STAT3, thus the finding was that the PD-1 protein is an important target for immune modulation (52, 53). The present study showed the most significant alterations in pathways related to lipid metabolism in the GLPP group, such as the synthesis and catabolism of fatty acids (glycerophospholipid, hexadecanoic acid, oleic acid, and glycerophospholipid). However, the LPCs (16:0), LPCs (17:0), LPCs (18:0), LPCs (20:1), glycerophosphatidylcholine, and sn-glycerol-3-phosphatidylcholine were significantly upregulated in the LMS group, which indicated that LMS caused severe disorders of glycerophospholipid metabolism in the body. Thus, the immunoregulation of GLPP may affect the signaling pathways of lipid metabolism through the PD-1 protein, while LMS alters the levels of LPCs *in vivo*, which act as inflammatory factors and control endothelial cell proliferation and apoptosis in immune regulation (54).

In addition to PD-1, AA is related to immune and anti-inflammatory effects (55, 56). The present study found that compared with the CK group, AA was significantly downregulated, the 12-hete as a metabolite of AA was upregulated in the CTX group, AA was returned to normal by the treatment of GLPP and LMS, and the 12-hetes were significantly down-regulated in the GLPP group. LTD4 is produced from free AA via the lipoxygenase (LOX) action pathway (57). Our data found that LTD4 was significantly upregulated in the GLPP and LMS groups, suggesting that GLPP and LMS altered the AA metabolic pathway. Oleic and linoleic acids are metabolized to AA in the body (58). In the present study, compared with the CK group, oleic acid in the CTX group was significantly upregulated and significantly downregulated after GLPP treatment, which further suggests that GLPP treatment alleviates the abnormal metabolism of AA, which was consistent with the results of increased oleic acid content in CTX-treated mice by sulfated *Mesona chinensis* polysaccharide (59).

The cAMP pathway is associated with many endocrine-related pathways, including cortisol (ketone) synthesis and secretion, fatty acid degradation, apoptosis, and tumor-related signal transduction pathways (60, 61). It was found that *G. lucidum* polysaccharides could inhibit tumor growth through the cAMP-PKA signaling pathway, activating host immune function (62). Therefore, it can be speculated that the regulation of the immune system by GLPP in CTX-induced immunosuppression mice may be related to cAMP and its downstream pathways through GLPP as a second messenger, but more details need to be further investigated and explored.

## 5. Conclusion

*Ganoderma lucidum* polysaccharide peptide is an important component of *G.lucidum* and is famous for enhancing the body's immunity. The research aimed to systematically investigate the effects of GLPP on CTX-induced immune damage in the organism and the molecular mechanisms involved. The results confirmed that GLPP was effective in protecting immune organs and enhancing the production of immune-related cytokines. Metabolite markers in the intestine, such as AA, LTD4, indole-3-ethanol, and CF, were adjusted, and citric acid, malic acid, cortisol, and oleic acid were revised to normal levels by GLPP to enhance the immune effect *in vivo*. Furthermore, some pathways associated with the immune system, such as the folate cycle, methionine cycle, TCA cycle, fatty acid biosynthesis and metabolism, glycerophospholipid metabolism, AA metabolism, and cAMP, were related to GLPP. These findings provide a theoretical basis for GLPP to be comprehensively utilized as an immunoreactive substance.

## Data availability statement

The original contributions presented in the study are included in the article/supplementary material, further inquiries can be directed to the corresponding authors.

## Ethics statement

The animal study was reviewed and approved by No. FJMU IACUC 2019-0084.

## Author contributions

JX: data curation, writing—original draft, and writing—review and editing. DL: critical content review and investigation. JL: software and validation. TZ: investigation and visualization. SL: conceptualization and methodology. ZL: methodology and supervision. All authors contributed to the article and approved the submitted version.
